# Inbreeding depression is associated with recent homozygous-by-descent segments in Belgian Blue beef cattle

**DOI:** 10.1186/s12711-024-00878-7

**Published:** 2024-01-31

**Authors:** Maulana Mughitz Naji, José Luis Gualdrón Duarte, Natalia Soledad Forneris, Tom Druet

**Affiliations:** 1 Unit of Animal Genomics, GIGA-R & Faculty of Veterinary Medicine, University of Liège, Quartier Hôpital, Avenue de l’Hôpital, 11, 4000 Liege, Belgium; 2 Walloon Breeders Association (awe groupe), 5590 Ciney, Belgium

## Abstract

**Background:**

Cattle populations harbor generally high inbreeding levels that can lead to inbreeding depression (ID). Here, we study ID with different estimators of the inbreeding coefficient *F*, evaluate their sensitivity to used allele frequencies (founder versus sample allele frequencies), and compare effects from recent and ancient inbreeding.

**Methods:**

We used data from 14,205 Belgian Blue beef cattle genotyped cows that were phenotyped for 11 linear classification traits. We computed estimators of *F* based on the pedigree information (*F*_PED_), on the correlation between uniting gametes (*F*_UNI_), on the genomic relationship matrix (*F*_GRM_), on excess homozygosity (*F*_HET_), or on homozygous-by-descent (HBD) segments (*F*_HBD_).

**Results:**

*F*_UNI_ and *F*_GRM_ were sensitive to used allele frequencies, whereas *F*_HET_ and *F*_HBD_ were more robust. We detected significant ID for four traits related to height and length; *F*_HBD_ and *F*_UNI_ presenting the strongest associations. Then, we took advantage of the classification of HBD segments in different age-related classes (the length of an HBD segment being inversely related to the number of generations to the common ancestors) to determine that recent HBD classes (common ancestors present approximately up to 15 generations in the past) presented stronger ID than more ancient HBD classes. We performed additional analyses to check whether these observations could result from a lower level of variation in ancient HBD classes, or from a reduced precision to identify these shorter segments.

**Conclusions:**

Overall, our results suggest that mutational load decreases with haplotype age, and that mating plans should consider mainly the levels of recent inbreeding.

**Supplementary Information:**

The online version contains supplementary material available at 10.1186/s12711-024-00878-7.

## Background

Mating of individuals sharing common ancestors results in inbreeding, a process associated with deleterious effects such as recessive genetic defects [1] or inbreeding depression (ID) that refers to the reduction of fitness observed in inbred individuals [2, 3]. Inbreeding is common in livestock species e.g. [4], as a consequence of a reduced effective population size (N_e_) and intensive use of superior breeders, but also in wild endangered species [2]. At the individual level, inbreeding is quantified through the inbreeding coefficient *F*, commonly defined as the probability that, at a given locus, the two alleles from a diploid individual are identical-by-descent [5]. In the past, inbreeding coefficients have been estimated mainly with the available genealogy but it has been shown that genomic estimators, obtained with genotyping or sequence data, better capture realized inbreeding as long as the number of markers is sufficiently large [6]. Several genomic estimators of the inbreeding coefficient have been proposed and have been compared in several studies, e.g. [7–9], without a clear consensus on which is best. Nevertheless, recent studies tend to show that the estimators based on the correlation between uniting gametes or on the proportion of the genome in runs-of-homozygosity (ROH) perform well [10, 11]. Importantly, these studies showed that the optimal coefficient might depend on the effective population size and the population demographic history.

Runs-of-homozygosity are long stretches of homozygous genotypes within individual genomes and are used as proxies for homozygous-by-descent (HBD) segments (chromosomal segments inherited twice from a common ancestor without recombination). Alternatively, model-based approaches using allele frequencies (AF), the genetic map and probabilities of genotyping errors have been developed to estimate HBD probabilities [12–15]. These methods, based on hidden Markov models (HMM), have been extended to work with sequence data (genotype probabilities or allele counts) and are particularly useful with heterogeneous or degraded information (e.g. low marker density, low minor allele frequency (MAF), variable marker spacing or low coverage). The length of the HBD segments is a function of the number of generations to the common ancestor, e.g. [16], as more generations result in more opportunities to break the segments through recombination. The distribution of their length is thus informative on past demographic events, such as the timing of inbreeding events, as illustrated in Kirin et al. [17], Pemberton et al. [18] or Ceballos et al. [19]. The relationship between length of HBD segments and number of generations to the common ancestor allows to test whether longer segments, associated with more recent common ancestors, are more deleterious. Such an approach has been applied in cattle by Doekes et al. [20] and Makanjuola et al. [21], without strong and consistent evidence indicating that longer ROH are more significantly associated to ID. More recently, the authors from a similar study conducted on wild Soay sheep concluded that mutation load decreases with haplotype age [22].

The first objective of the present study was to make an empirical comparison of different estimators of inbreeding coefficients to confirm observations from previous studies, often relying on simulated data. To that end, we studied ID in 11 traits related to body size or muscular development measured in a large cohort of genotyped cows from the Belgian Blue beef cattle population. In addition, using traits presenting significant ID, we investigated whether recent HBD segments were more deleterious than ancient ones.

## Methods

### Data

We performed our study with the data used by Gualdrón Duarte et al. [23]. Briefly, the data consisted in a cohort of 14,762 Belgian Blue beef cows genotyped for a set of 28,858 single nucleotide polymorphisms (SNPs), after filtering for call rate (> 0.95), Hardy–Weinberg equilibrium (p > 0.001) and MAF (> 0.01). For 11,521 cows, genotypes were obtained by imputation from low-density arrays. The MAF threshold was applied because imputation is less accurate for rare alleles, although rare alleles can be informative for estimating HBD probabilities. The individuals had trait deviations (i.e., phenotypes corrected for fixed effects from the evaluation model) available for 11 linear classification traits related to muscular development and body size: top muscling, shoulder muscling, buttock muscling (rear and side view), rump, rib shape, chest width, length, pelvis length, pelvis width and stature. The raw phenotypes are described in Additional file 1: Table S1, the classification scale going from 1 to 50. We selected cows born between 2011 and 2019, resulting in 14,205 individuals. Among these, 12,360 were phenotyped for stature and 13,926 had phenotypes for the other ten linear scores.

### Estimation of the inbreeding coefficient *F*

The inbreeding coefficients *F* were estimated using six approaches including the pedigree-based *F*_PED_ obtained using a pedigree containing 60,454 individuals (average pedigree depth = 10.6), the estimator based on the excess of homozygosity *F*_HET_ from Li and Horvitz [24] and estimated using the --het option from PLINK [25], the estimator based on the correlation between uniting gametes *F*_UNI_ [24, 26] obtained from GCTA [26], the estimator based on the diagonal elements of the genomic relationship matrix (GRM) *F*_GRM_ computed with GCTA and using the first (*F*_GRM-1_) and the second (*F*_GRM-2_) rules proposed by VanRaden [27], and an estimator based on the proportion of the genome lying in HBD segments, *F*_HBD_. To that end, we estimated HBD probabilities with the model-based approach from Druet and Gautier [15] implemented in the RZooRoH R-package [28]. In this hidden Markov model (HMM), the genotypes, allele frequencies (AF), genotyping errors rates and the genetic map are used to model the genome as a mosaic of HBD and non-HBD segments. Multiple HBD classes are defined, and in class *c*, the length of HBD segments is exponentially distributed with rate *R*_*c*_ (i.e., the expected length is equal to 1/*R*_*c*_ Morgan). Thus, each class corresponds to a different group of ancestors that were present approximately 0.5 *R*_*c*_ generations in the past (see [15]). Here, these HBD classes are modelled as successive layers of ancestors (setting layers = TRUE in the model) as described in Druet and Gautier [29]. We fitted a model with nine HBD classes with rates *R*_c_ = {2, 4, 8, …, 512} and one non-HBD class. Finally, *F*_HBD_ was estimated as the proportion of the genome in HBD classes with *R*_c_ ≤ 256 because fewer SNPs are expected per segment in the last HBD class (the estimated HBD proportion is then more equivalent to a SNP-by-SNP maximum likelihood estimator, relying more on AF - see [10]). The model provides also the proportion of the genome in each HBD class *c*, *F*_HBD-*c*_. More details on the different estimators can be found for instance in Alemu et al. [10].

We estimated the AF in the base generation of the pedigree with the gene content approach proposed by Gengler et al. [30]. In this approach, founder AF are estimated separately for each marker. To do this, we defined the individual allele dosages as phenotypes. More precisely, the vector ${\mathbf{z}}$ of gene content for a marker contains the number of reference alleles observed for each individual (e.g. 0, 1 and 2 for genotypes AA, AB and BB, respectively). This vector is then modelled as ${\mathbf{z}} = \mathbf{1}\mu + {\mathbf{Wu}} + {\mathbf{e}}$, where $\mu$ is the expected gene content in the base population (equal to 2*f*_i_, where *f*_i_ is the founder allele frequency at marker *i*), ${\mathbf{u}}$ is a vector of individual deviations from the expected gene content and ${\mathbf{e}}$ is a vector of error terms equal to 0 in the absence of genotyping errors. The **u** vector is expanded to include all individuals in the pedigree, with ${\text{var}}\left( {\mathbf{u}} \right) = {\mathbf{A}}{\upsigma }_{{\text{u}}}^{2}$. This mixed model was solved using a pedigree-BLUP with blupf90 [31], with a heritability of 0.99 to account for genotyping errors. Gengler et al. [30] showed that the AF in the reference population can be estimated as the mean effect from the model divided by two (SNPs with an estimated MAF lower than 0.01 were filtered out).

### Estimation of the ID and stratification of ID by age-related HBD classes

Inbreeding depression was estimated for each trait and using the six inbreeding coefficients *F* with the following linear mixed model with GCTA [26]:

$$y_{i} = \mu + u_{i} + b\;F_{i} + e_{i } ,$$

where $y_{i}$ is the trait deviation for the $i$th individual, $u_{i}$ is its random polygenic effect. The vector of the polygenic effects is $\sim N\left( {{\mathbf{0}},{\mathbf{G}}\sigma_{g}^{2} } \right)$, with ${\mathbf{G}}$ being the GRM and $\sigma_{g}^{2}$ the additive genetic variance. $F_{i}$ is the inbreeding coefficient for the $i$th individual, $e_{i}$ is its residual error, $\mu$ is the mean effect and $b$ is the ID effect (the effect associated with an inbreeding level equal to 1). The genetic relationship matrix was selected according to the fitted *F*: we used the pedigree-based additive relationship matrix ${\mathbf{A}}$ with *F*_ped_, the GRM obtained with the first rules defined by VanRaden [27] for *F*_UNI_ and *F*_GRM-1_ and with the second rules for *F*_GRM-2_, and a similarity matrix obtained by setting AF to 0.5 for *F*_HET_ and *F*_HBD_. The significance thresholds were set at p < 7.6e−4 to account for multiple testing for 66 independent tests (this is a conservative threshold as both traits and *F* estimators are not completely independent).

For traits presenting significant ID, we subsequently applied a model that fits simultaneously the proportion of the genome in the eight HBD classes (*R*_c_ = {2, 4, 8, …, 256}). This allows to compare their contribution to ID and to test whether some classes are more deleterious:

$$y_{i} = \mu + u_{i} + \sum \limits_{c = 1}^{8} b_{c} F_{HBD{\text{-}}c, i} + e_{i} ,$$

where *F*_*HBD-c,i*_ is the proportion of genome in HBD class $c$ for the $i$th individual, and $b_{c}$ is the effect associated to inbreeding levels in the corresponding class.

### Validation of the approach to stratify ID according to age-related HBD classes

The estimated values of $b_{c}$ and their significance level might be influenced by properties of the data and do not reflect only biological differences between HBD classes. Indeed, the ability of the model to estimate the effect associated with each HBD class depends on the level of variation within each class (e.g., we cannot estimate the effect in a class without variation). Another potential issue is related to the accuracy of estimated proportions of the genome lying in different HBD classes. For instance, these proportions might be estimated more accurately for recent HBD classes associated with long segments containing more SNPs. If *F*_HBD-*c*_ are less accurate for ancient HBD classes, we might expect to have less power to detect their effect.

To address the first problem associated with different levels of variation in different HBD classes, we relied on a simple simulation approach. We used the available genotypes for the 28,858 markers to simulate a polygenic architecture. For each SNP, the additive locus effect was obtained by multiplying the allele dosage by the allelic effect, which was randomly drawn from a standard normal distribution. The polygenic effect was obtained as the sum of all additive locus effects, while residual error terms were randomly sampled from a standard normal distribution. The polygenic and residual variances were then adjusted to match the heritability and phenotypic variance of stature. Then, we simulated an overall ID effect equal to − 21 (the regression coefficient obtained for *F*_HBD_ using the real stature phenotypes—see “Results”). This was achieved by multiplying the estimated values of *F*_HBD_ by − 21, thus assuming a constant ID effect across HBD classes. The effect of inbreeding on the phenotype of individual $i$ is therefore equal to its inbreeding coefficient (*F*_HBD,*i*_) multiplied by − 21. Individual phenotypes were finally calculated by summing the polygenic effect, the error term and the effect associated with ID. Then, we estimated ID on the simulated phenotypes using the same linear mixed model as described above and repeated 100 simulations. This approach simply tests whether the level of variation allows the contribution of each class to be captured, but does not take errors in the estimation of *F*_HBD-*c*_ into account.

To address the second potential issue, we took advantage of the available imputed genotypes for 572,667 SNPs from the Illumina BovineHD array (from the study from Gualdrón Duarte et al. [23]). This represents a 20-fold increase in marker density and should allow more accurate estimation of HBD proportions in different classes, particularly for those with higher rates. Thus, we applied the multiple HBD class model with 11 HBD classes with *R*_c_ = {2, 4, 8, 16, …, 2048} and one non-HBD class, and fitted the same linear mixed model as described above. The model was extended to 11 HBD classes because the marker density now allows shorter HBD segments to be captured. We repeated the simulation study with this second dataset.

## Results

### Impact of allele frequencies on estimated inbreeding coefficients

Ideally, AF from the reference population should be used to estimate inbreeding coefficients with methods that require such information (i.e. *F*_UNI_, *F*_GRM-1_, *F*_GRM-2_ and *F*_HBD_). However, this is rarely done as these values are unknown and AF from the current population are used instead. The correlation between estimators obtained with base population AF versus sample AF were equal to 0.86, 0.66, 0.46 and 0.99 for *F*_UNI_, *F*_GRM-1_, *F*_GRM-2_ and *F*_HBD_, respectively, indicating that the first three estimators were more sensitive to these values. Although *F*_HBD_ is robust to changes in used AF, correlations between HBD proportions in different HBD classes (*F*_HBD-*c*_) indicate that the AF have little influence for recent HBD classes associated with long segments with many markers (see Additional file 1: Table S2). The correlations were indeed higher than 0.99 for HBD classes with rates *R*_c_ ≤ 16 (and higher than 0.94 for HBD classes with rates *R*_c_ ≤ 64), but dropped to between 0.80 and 0.90 for the last three HBD classes indicating that these are more influenced by AF. When *F*_UNI_, *F*_GRM-1_ and *F*_GRM-2_ were estimated with the sample AF, they exhibited a different trend of annual rates of inbreeding, estimated per year of birth, over the 2011–2019 period (respectively + 0.00, − 0.01 and − 0.02) compared to the trend estimated with the three other estimators (+ 0.01—see Additional file 2: Fig. S1). With the reference population AF, trends were equal to + 0.01 with all estimators. In agreement, correlations between the different estimators were lower when sample AF were used instead of reference population values (see Additional file 1: Table S3). For instance, the correlations between *F*_GRM-1_ and *F*_PED_, *F*_HET_ and *F*_HBD_ increased from respectively − 0.16, 0.39 and 0.39 to 0.28, 0.79 and 0.77. The same values changed from − 0.30, 0.11 and 0.13 to 0.24, 0.62 and 0.61 for *F*_GRM-2_, and from 0.11, 0.78 and 0.75 to 0.36, 0.94 and 0.90 for *F*_UNI_. Thus, hereafter, we will use estimated AF from the reference population.

### Inbreeding depression for traits related to body dimensions and muscular development

Significant ID was found for four traits: stature, length, pelvis length and pelvis width (Table 1). Significant values were obtained for these traits with most of the estimators of *F*, but evidence was always lower when using *F*_PED_. The strongest effects and associations were observed for stature, length and pelvis length (e.g., p < 1e−12 with *F*_HBD_). For these traits, the lowest p-values were achieved with *F*_HBD_ followed by *F*_UNI_.

**Table 1:** Estimation of inbreeding depression for 11 traits with six distinct estimators of *F* (base population AF were used)

Traits	Phenotyped animals	*F*_PED_		*F*_UNI_		*F*_GRM-1_		*F*_GRM-2_		*F*_HET_		*F*_HBD_	
		Effect	p-values	Effect	p-values	Effect	p-values	Effect	p-values	Effect	p-values	Effect	p-values
Buttock muscling (rear view)	13,926	0.12	9.1e−01	− 0.81	1.0e−01	− 1.22	9.9e−03	− 1.19	1.2e−02	− 0.51	2.8e−01	− 0.18	7.4e−01
Buttock muscling (side view)	13,926	0.18	8.7e−01	− 0.42	4.0e−01	− 0.76	1.1e−01	− 0.84	7.8e−02	− 0.07	8.8e−01	0.23	6.8e−01
Chest width	13,926	3.25	1.3e−01	0.72	5.0e−01	− 0.21	8.3e−01	− 0.43	6.6e−01	1.40	1.6e−01	2.12	6.4e−02
Length	13,926	− 4.71	5.3e−04	− 5.10	3.5e−15	− 4.33	2.6e−12	− 4.33	2.2e−12	− 4.86	4.0e−15	− 5.60	2.3e−15
Pelvis length	13,926	− 4.03	1.3e−03	− 4.37	1.2e−12	− 3.77	1.2e−10	− 3.66	3.2e−10	− 4.19	8.2e−13	− 4.73	1.5e−12
Pelvis width	13,926	− 0.54	5.8e−01	− 2.04	1.3e−05	− 2.20	7.7e−07	− 2.17	8.7e−07	− 1.58	3.7e−04	− 1.48	3.5e−03
Rib shape	13,926	4.90	7.6e−03	− 0.04	9.7e−01	− 0.59	4.9e−01	− 0.40	6.4e−01	0.30	7.2e−01	0.16	8.7e−01
Rump	13,926	0.37	8.1e−01	0.87	2.5e−01	0.53	4.7e−01	0.10	8.9e−01	1.17	1.1e−01	1.61	5.2e−02
Shoulder muscling	13,926	0.20	8.8e−01	− 0.55	3.8e−01	− 1.14	5.9e−02	− 1.13	6.1e−02	− 0.12	8.4e−01	0.06	9.3e−01
Stature	12,360	− 19.84	3.9e−04	− 18.16	7.8e−13	− 16.36	1.5e−11	− 16.67	8.7e−12	− 17.38	8.5e−13	− 20.55	1.4e−13
Top muscling	13,926	1.95	4.5e−01	− 1.42	2.4e−01	− 2.10	6.6e−02	− 2.06	7.2e−02	− 0.73	5.2e−01	− 0.46	7.3e−01

For each test, we report the estimated effect and the significance level

**Fig. 1:**
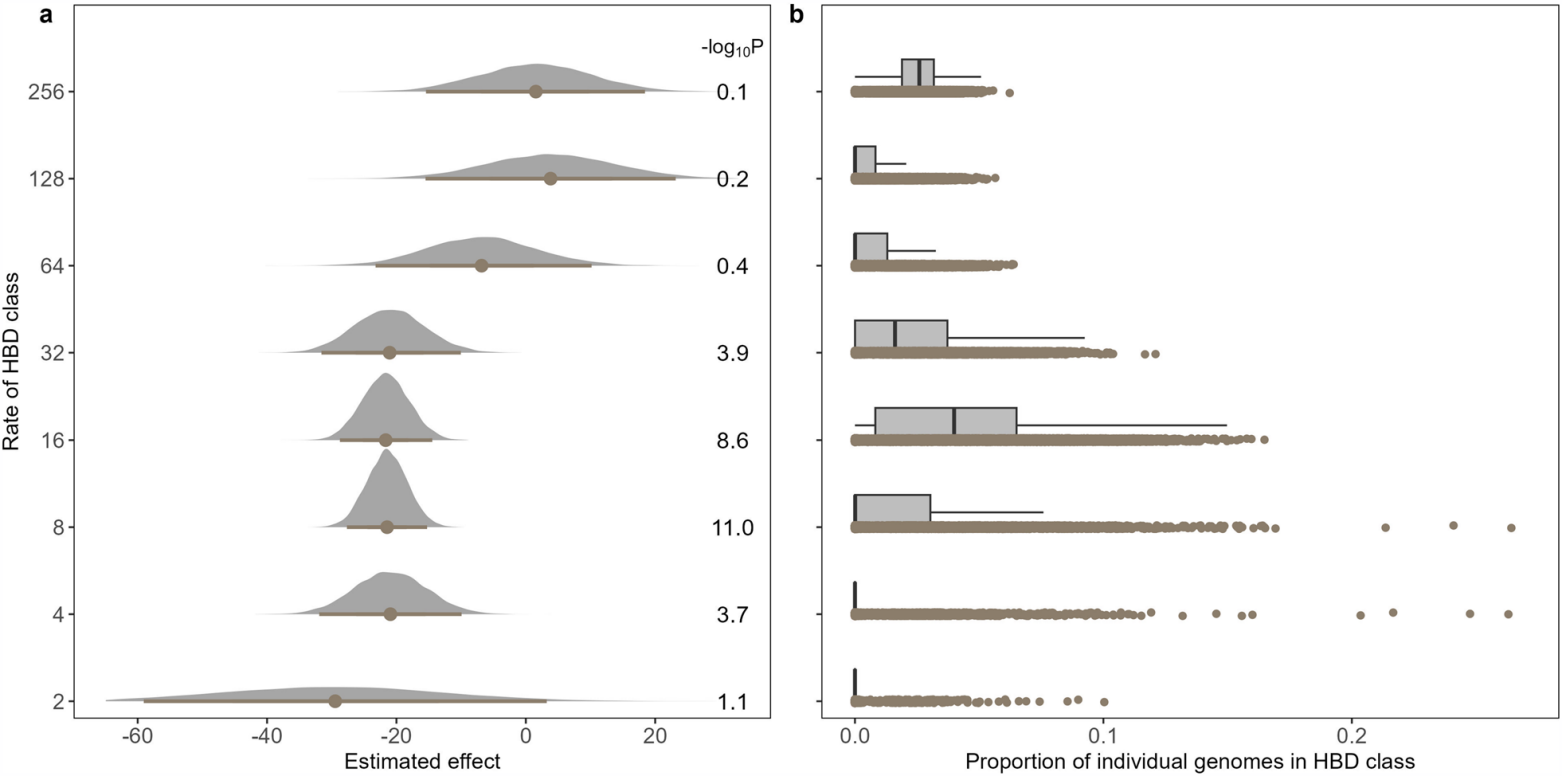
Inbreeding depression associated with different HBD classes. **a** Estimated inbreeding depression effects (and confidence intervals) per class for stature; **b** distribution of autozygosity levels per HBD class. Partitioning of autozygosity in different HBD classes was performed with medium-density genotyping data (~ 30K SNPs). The values on the right of **a** represent the strength of association (p-values on a −log10 scale)

Then, we estimated the ID associated with different HBD classes (Fig. 1a) and observed stronger effects for more recent HBD classes (longer HBD segments). For the classes with *R*_*c*_ < 64, the estimated effects were < − 20, these values decreased to − 6.75 for the class with *R*_*c*_ = 64, and dropped to around 0 for the two most ancient fitted classes. In addition, the estimated effects were not significantly different from 0 for classes with *R*_*c*_ ≥ 64. However, we also observed that HBD classes with lower levels of variation (Fig. 1b) presented less significant p-values. Similar patterns were observed for other traits (see Additional file 2: Fig. S2).

### Simulation study and analysis with a higher marker density

**Fig. 2:**
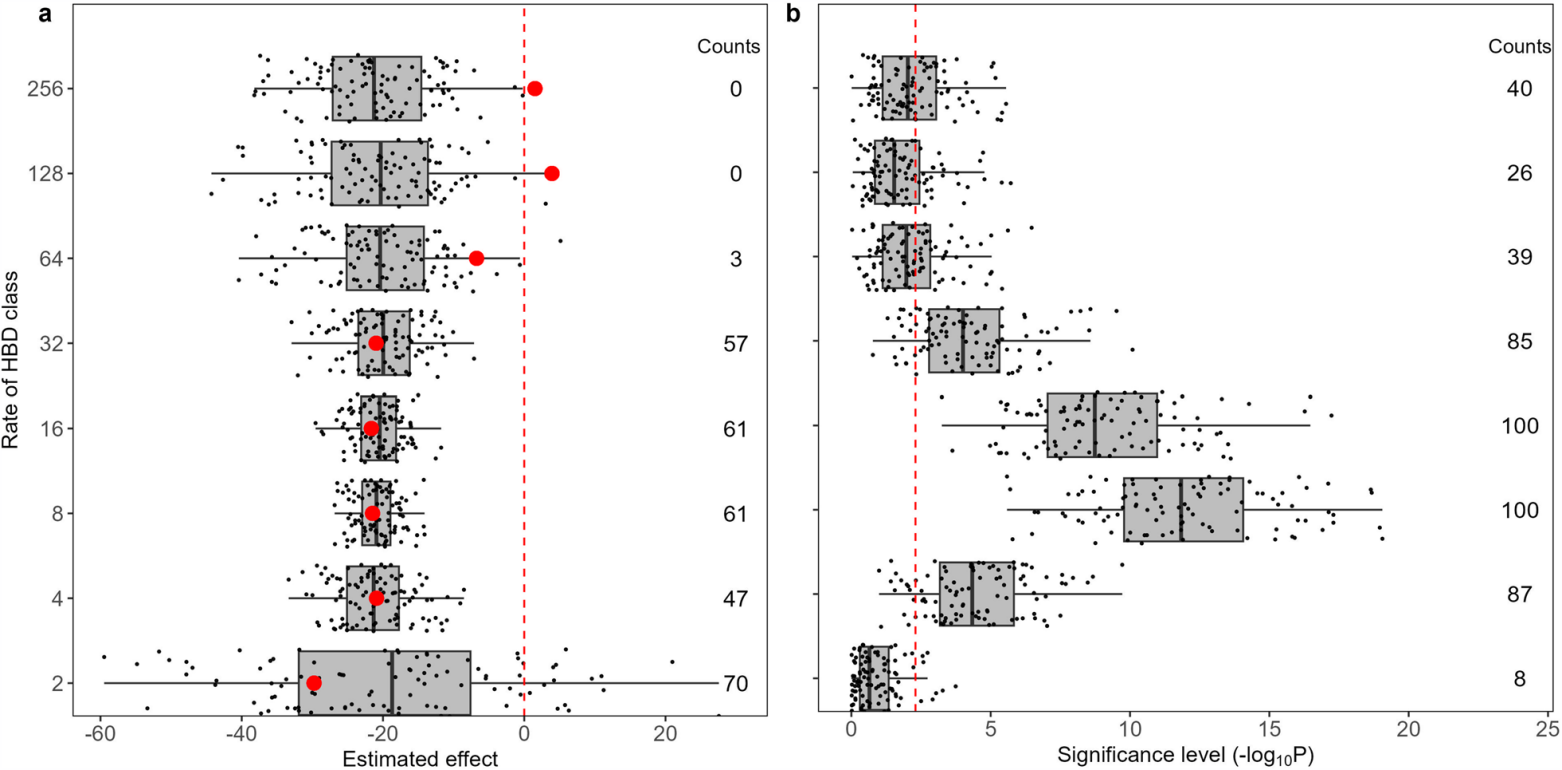
Estimated inbreeding effects (**a**) and associated significance levels (**b**) for 100 simulations. Each replicate was simulated under the assumption that all HBD classes have the same effect (− 21). Partitioning of autozygosity in different HBD classes was performed with medium-density genotyping data (~ 30K SNPs). The counts on the right of each panel represent the number of simulations with higher effects than the value estimated in the real data (**a**) or the number of simulations with significant association (**b**)

We conducted additional analyses to assess whether the lower significance levels observed for ancient HBD classes and the stronger ID effects estimated for more recent HBD classes could be due to the lower informativeness of our dataset for estimating ancient HBD levels. First, we compared our results with analyses on simulated data with constant levels of ID across classes and with the same structure as in our real dataset (i.e. with identical levels of variation in different HBD classes) (Fig. 2). Compared to the value estimated with the real data, the estimated ID effects for the HBD class with *R*_*c*_ equal to 64 were always stronger in the simulations (more negative and deleterious effects on the phenotypes), except for three simulations, while this was always true for the estimated ID effects for the two most ancient HBD classes (*R*_*c*_ equal to 128 and 256). For the other HBD classes, the estimated ID values were more in line with the values obtained in the simulations. As expected, the power to detect ID was lower in HBD classes with less variation (ancient HBD classes, but also the most recent HBD class with *R*_*c*_ = 2). Significant ID effects were detected in 39, 26 and 40% of the simulations for classes with rates of 64, 128 and 256, respectively. To note, significant ID effects were observed in 54% of the simulations for at least one of these three HBD classes. For the other HBD classes (*R*_*c*_ = {4, 8, 16, 32}), significant ID effects were detected in more than 85% of the simulations (100% for the classes with rates 8 and 16). Overall, these results indicate that there is less power to detect ID effects associated with more ancient HBD classes, which have lower levels of variation. Nevertheless, in more than 50% of the simulations where ID is assumed to be constant across all HBD classes, we detected significant ID effects in at least one of the three more distant HBD classes, and the estimated ID effects were generally more deleterious than those obtained on the real data for the ancient HBD classes. This suggests that in the real data, ID effects are not constant across HBD classes, but rather that ancient HBD classes are likely to be less deleterious than more recent HBD classes.

**Fig. 3:**
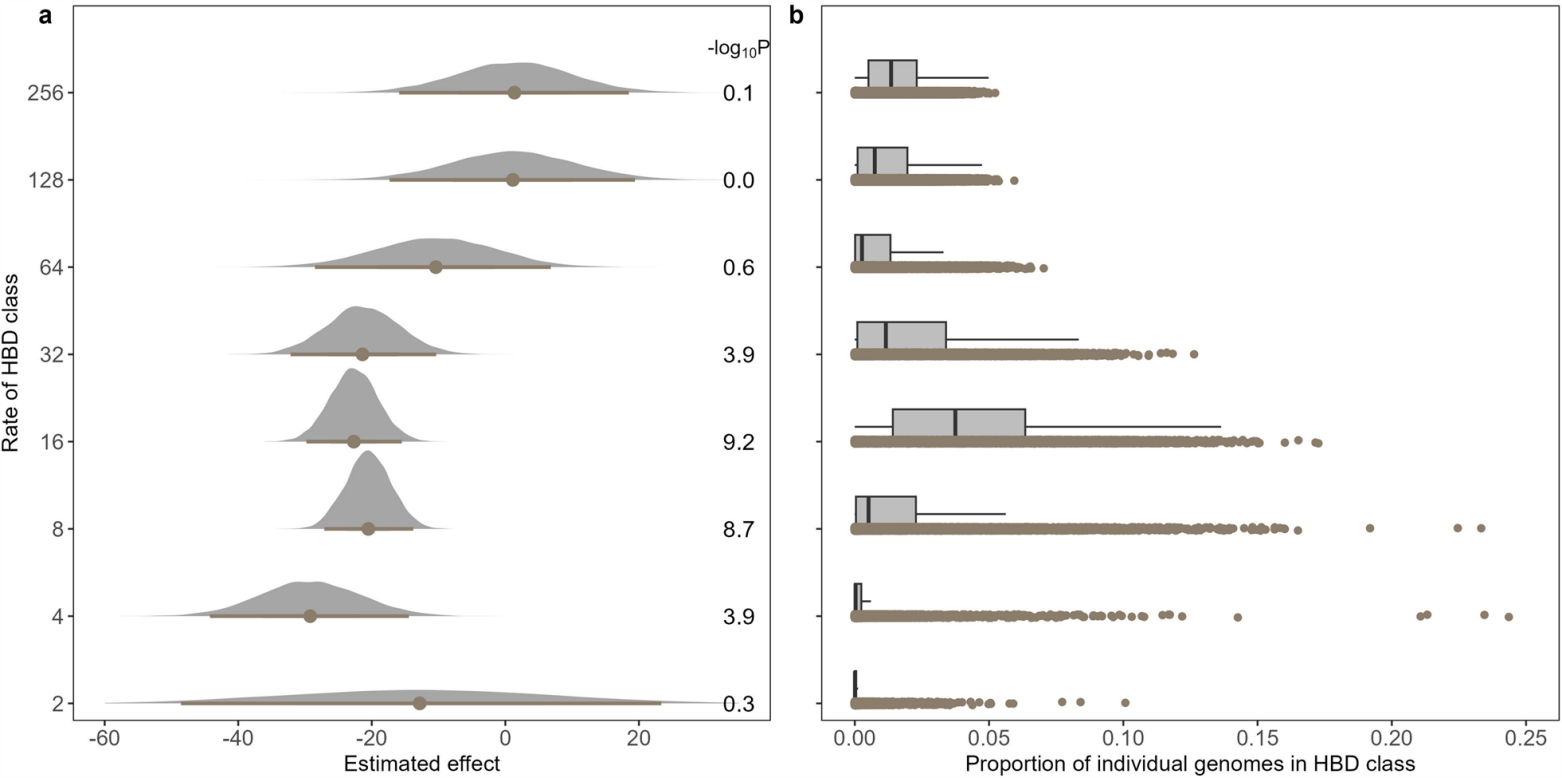
Inbreeding depression associated with different HBD classes. **a** Estimated inbreeding depression effects (and confidence intervals) per class for stature; **b** distribution of autozygosity levels per HBD class. Partitioning of autozygosity in different HBD classes was performed with high-density genotyping data (~ 567K SNPs). The values on the right of **a** represent the strength of association (p-values on a −log10 scale)

Next, we repeated the analysis with a higher marker density using imputed genotypes. The results were in line with those obtained at lower density levels (Fig. 3 for stature and see Additional file 2: Fig. S3 for other traits), with less deleterious effects associated with more ancient inbreeding. Effects were close to zero (and non-significant) for ancient HBD classes whereas large significant deleterious effects were estimated for HBD classes with rates from 2 to 32 (with lower significance for the most recent class). As before, the HBD class with *R*_*c*_ = 64 presented intermediate values. This analysis suggests that the lower estimated effects are not due to a smaller number of markers per segment for the ancient classes. Simulations with this second set of genotypes were in agreement with the first simulations (see Additional file 2: Fig. S4).

## Discussion

As recently shown by Caballero et al. [32], when founder AF are used, estimators of the inbreeding coefficient present better properties as illustrated through more consistent trends through years, higher correlations between different estimators, and also estimated levels of ID (see below). For instance, correlations between *F*_GRM-1_ and *F*_PED_ or *F*_HBD_ were much higher compared to values reported by Solé et al. [33] in the same population. Conversely, using the current population AF can lead to very different estimators, in particular when AF are evolving fast due to high drift (i.e., small effective population size) or high selection levels, which both typically occur in livestock populations. For instance, cattle populations typically present values of N_e_ around 100 and harbor large selective sweeps that reflect rapid changes in AF [34]. Nevertheless, base population AF are not always available. They require either ancient samples (genotypes from the base population) or need to be estimated with statistical models relying either on the pedigree of the genotyped animals [30], or on genotyped samples from different populations that diverged [35]. Such information is not always available and in the best cases, such approaches will allow to obtain AF only for a relatively close base population. When these AF remain unknown, it is better to use methods that are less sensitive to their values. These methods include for instance the simple homozygosity measure. We showed here that the longest HBD segments (i.e., the most recent HBD classes) are little impacted by the selected AF whereas more ancient classes (short segments with few SNPs) are more sensitive, and should not be systematically included in the estimator of *F*.

Inbreeding depression was detected for four traits when using the genomic estimators, and for two traits when using *F*_ped_, which achieved clearly lower significance levels compared to the genomic estimators. This indicates that the power of *F*_ped_ to detect ID is lower. However, genomic estimators capture more generations of inbreeding and this may be an advantage. If deeper genealogies could be used with the pedigree-based estimator, it may be more efficient and have also higher correlations with genomic estimators (although recent generations have the greatest contribution to variation in levels of inbreeding). The five genomic inbreeding coefficients presented relatively similar significance or ID levels, suggesting that when the base AF are available, the estimators have close properties as indicated by Caballero et al. [32].

Using the partitioning of HBD in different classes, we observed that ancient inbreeding was not associated to ID, in agreement with the observation that longer ROH are enriched in deleterious segments [36] or that deleterious alleles are younger than neutral ones [37], and with similar studies in livestock species [21] or in wild populations [22]. It is tempting to conclude that ancient HBD is thus less depressive, or that longer HBD tracks are enriched in deleterious mutations, as it matches the theory that deleterious mutations are young as they are continuously removed from the population through purifying selection [3]. Nevertheless, results must be interpreted with caution as we observed that ancient HBD classes presented less variation, and also because estimation of ancient HBD segments is less precise. Both these aspects could reduce the power to detect ID associated with more ancient classes, and are relevant for previous studies too. For instance, lower variation levels were also observed in the most ancient ROH class in Soay sheep [22]. Less variation is in fact expected for more ancient HBD classes as these correspond to the contribution of larger groups of ancestors. As the contributions are averaged over many lineages, they vary less than recent contributions that depend on a few genealogical branches. Using a higher marker density that was available for individuals from the same breed, Solé et al. [33] previously showed that although ancient classes contributed more to the total levels of autozygosity, they presented less variation as they reflected more the overall population history (common to all individuals). In addition, the recent evolution of past effective population size estimated on 634 Belgian Blue bulls with GONE [38] indicates that N_e_ has been low only in the recent past (see Additional file 2: Fig. S5). Larger N_e_ in more ancient generations, will further reduce the level of variation of associated autozygosity. This will be true in many livestock species presenting only a recent decrease in N_e_, e.g. [39, 40].

Consequently, we investigated whether the reduced accuracy or levels of variation in ancient HBD classes could influence the conclusion of similar studies. We observed that the power to detect ID was indeed reduced in more ancient classes, corresponding to ancestors that were present more than 30 generations ago. Although we could significantly detect the ID in some simulations, it remained frequently undetected. However, estimated effects were most often more pronounced (albeit non-significant) than in our real data. Results were also confirmed at higher marker density, allowing to estimate more accurately ancient HBD classes. Overall, the results show that ancient HBD seems to be less deleterious but also that results must be interpreted with caution and that additional and more powerful analyses should be designed.

From a pragmatic point of view, the most ancient HBD classes (> 30–50 generations) should not be included in the estimation of inbreeding levels used in different applications in livestock species. These ancient HBD segments might indeed be less deleterious as suggested by results from several studies that could not detect ID associated with shorter segments when working with standard genotyping arrays. At such marker densities, these classes contribute little to variation in inbreeding levels. Even if a higher marker density was available, these ancient HBD segments might not be relevant in management applications. Indeed, they trace back to many generations in the past, before the intensification of selection and reduction of N_e_. As a consequence, deleterious variants will have undergone a relatively long period of selection (including purifying). In addition, the true HBD levels in ancient classes are expected to present little variation as they correspond to many lineages tracing back to a period of larger N_e_. The optimal threshold to select HBD classes still remains to be defined. In our study, the HBD class associated with ancestors that were present 15 generations ago was still relevant, whereas the class corresponding to 50 generations in the past was clearly non-significant. The class that captured contributions from intermediary generations of ancestors (with rate equal to 64) had often lower estimated effects but was also sometimes significant (p < 0.05).

## Conclusions

The results of the present study confirm that founder AF should be used when estimating the inbreeding coefficient. In particular, estimators such as *F*_GRM_ or *F*_UNI_ are particularly affected by the AF used. When founder AF are not available, more robust estimators such as those based on HBD segments or ROH are recommended. We also found that ID is associated with recent HBD segments, suggesting that mutational load decreases with haplotype age. However, we showed that such findings should be interpreted with caution as there is less variation associated with ancient HBD segments and these are less accurately identified at intermediate marker density. Overall, our work indicates that mating plans should consider mainly the levels of recent inbreeding.

### Supplementary Information

**Additional file 1: Table S1.** Summary statistics for linear classifications traits in Belgian Blue Beef cattle. **Table S2.** Correlations between individual proportions of the genome in different HBD classes (*F*_HBD-c_) estimated with AF from base population or with AF from the sample. **Table S3.** Correlation between estimators of the inbreeding coefficient estimated using sample allele frequencies or base population allele frequencies.

**Additional file 2: Figure S1.** Annual trend for the average inbreeding levels by birth years (a) estimated with sample AF; (b) estimated with base population AF. **Figure S2.** Inbreeding depression associated with different HBD classes estimated for length, pelvis length and pelvis width (with 50K genotyping array). **Figure S3.** Inbreeding depression associated with different HBD classes estimated for length, pelvis length and pelvis width (with high-density genotyping array). **Figure S4.** Inbreeding effects and associated significance levels in 100 simulations. **Figure S5.** Recent evolution of past effective population size (N_e_) estimated in Belgian Blue cattle with GONE.

## Data Availability

The data that support the findings of this study are available from Elevéo and Inovéo (Awé Group, Belgium) but restrictions apply to the availability of these data, which were used under license for the current study, and thus are not publicly available.
