# Supplementary material for: Inbreeding depression is associated with recent homozygous-by-descent segments in Belgian Blue beef cattle
Source: Genet Sel Evol. 2024 Jan 31;56:10. doi: 10.1186/s12711-024-00878-7 (PMC10832232; doi:10.1186/s12711-024-00878-7)
Supplement: Supplementary file 1 — Additional file 1: Table S1. Summary statistics for linear classifications traits in Belgian Blue Beef cattle. Table S2. Correlations between individual proportions of the genome in different HBD classes (FHBD-c) estimated with AF from base population or with AF from the sample. Table S3. Correlation between estimators of the inbreeding coefficient estimated using sample allele frequencies or base population allele frequencies. [file 12711_2024_878_MOESM1_ESM.docx]

**Table S1.** Summary statistics for linear classifications traits in Belgian Blue Beef cattle

| Traits | Number records | Min. | Median | Mean | Max. | Variance | Stand. Dev. |
| --- | --- | --- | --- | --- | --- | --- | --- |
| Buttock muscling (rear view) | 14,476 | 3 | 38 | 38.2 | 47 | 2.9 | 1.71 |
| Buttock muscling (side view) | 14,476 | 12 | 38 | 38.4 | 47 | 3.3 | 1.81 |
| Chest width | 14,476 | 8 | 28 | 27 | 42 | 12.2 | 3.5 |
| Length | 14,476 | 20 | 39 | 39.3 | 49 | 4.8 | 2.19 |
| Pelvis length | 14,476 | 20 | 39 | 38.7 | 48 | 4.1 | 2.03 |
| Pelvis width | 14,476 | 11 | 38 | 38.4 | 45 | 2.6 | 1.61 |
| Rib shape | 14,476 | 10 | 27 | 25.9 | 38 | 8.1 | 2.85 |
| Rump | 14,476 | 10 | 25 | 24.7 | 41 | 5.9 | 2.43 |
| Shoulder muscling | 14,476 | 15 | 33 | 33.2 | 42 | 4.8 | 2.19 |
| Stature* | 12,904 | 51 | 80 | 80.4 | 100 | 76.8 | 8.76 |
| Top muscling | 14,476 | 2 | 28 | 27.8 | 42 | 17.1 | 4.13 |

*****Measured stature is transformed in a linear score from 51 to 100. For that, the difference with the reference height measured at the same age is multiplied by 2.5. This value is then increased by 75.

**Table S2.** Correlations between individual proportions of the genome in different HBD classes (*F*_HBD-c_) estimated with AF from base population or with AF from the sample.

| Rate of HBD class | Correlations |
| --- | --- |
| 2 | 1.00 |
| 4 | 0.99 |
| 8 | 0.99 |
| 16 | 0.99 |
| 32 | 0.98 |
| 64 | 0.94 |
| 128 | 0.88 |
| 256 | 0.84 |
| 512 | 0.82 |

**Table S3.** Correlation between estimators of the inbreeding coefficient estimated using sample allele frequencies (upper diagonal / red) or base population allele frequencies (lower diagonal / green). Diagonal elements (in blue) represent correlations between inbreeding coefficients estimated with the same method but with different allele frequencies.

|  | *F*_PED_ | *F*_UNI_ | *F*_GRM-1_ | *F*_GRM-2_ | *F*_HET_ | *F*_HBD_ |
| --- | --- | --- | --- | --- | --- | --- |
| *F*_PED_ | 1.00 | 0.11 | -0.16 | -0.30 | 0.38 | 0.41 |
| *F*_UNI_ | 0.36 | 0.86 | 0.87 | 0.70 | 0.78 | 0.75 |
| *F*_GRM-1_ | 0.28 | 0.94 | 0.66 | 0.95 | 0.39 | 0.39 |
| *F*_GRM-2_ | 0.24 | 0.84 | 0.95 | 0.46 | 0.11 | 0.13 |
| *F*_HET_ | 0.38 | 0.94 | 0.79 | 0.62 | 1.00 | 0.94 |
| *F*_HBD_ | 0.44 | 0.90 | 0.77 | 0.61 | 0.94 | 0.99 |
