# Supplementary material for: Inbreeding depression is associated with recent homozygous-by-descent segments in Belgian Blue beef cattle
Source: Genet Sel Evol. 2024 Jan 31;56:10. doi: 10.1186/s12711-024-00878-7 (PMC10832232; doi:10.1186/s12711-024-00878-7)
Supplement: Supplementary file 2 — Additional file 2: Figure S1. Annual trend for the average inbreeding levels by birth years (a) estimated with sample AF; (b) estimated with base population AF. Figure S2. Inbreeding depression associated with different HBD classes estimated for length, pelvis length and pelvis width (with 50K genotyping array). Figure S3. Inbreeding depression associated with different HBD classes estimated for length, pelvis length and pelvis width (with high-density genotyping array). Figure S4. Inbreeding effects and associated significance levels in 100 simulations. Figure S5. Recent evolution of past effective population size (Ne) estimated in Belgian Blue cattle with GONE. [file 12711_2024_878_MOESM2_ESM.docx]

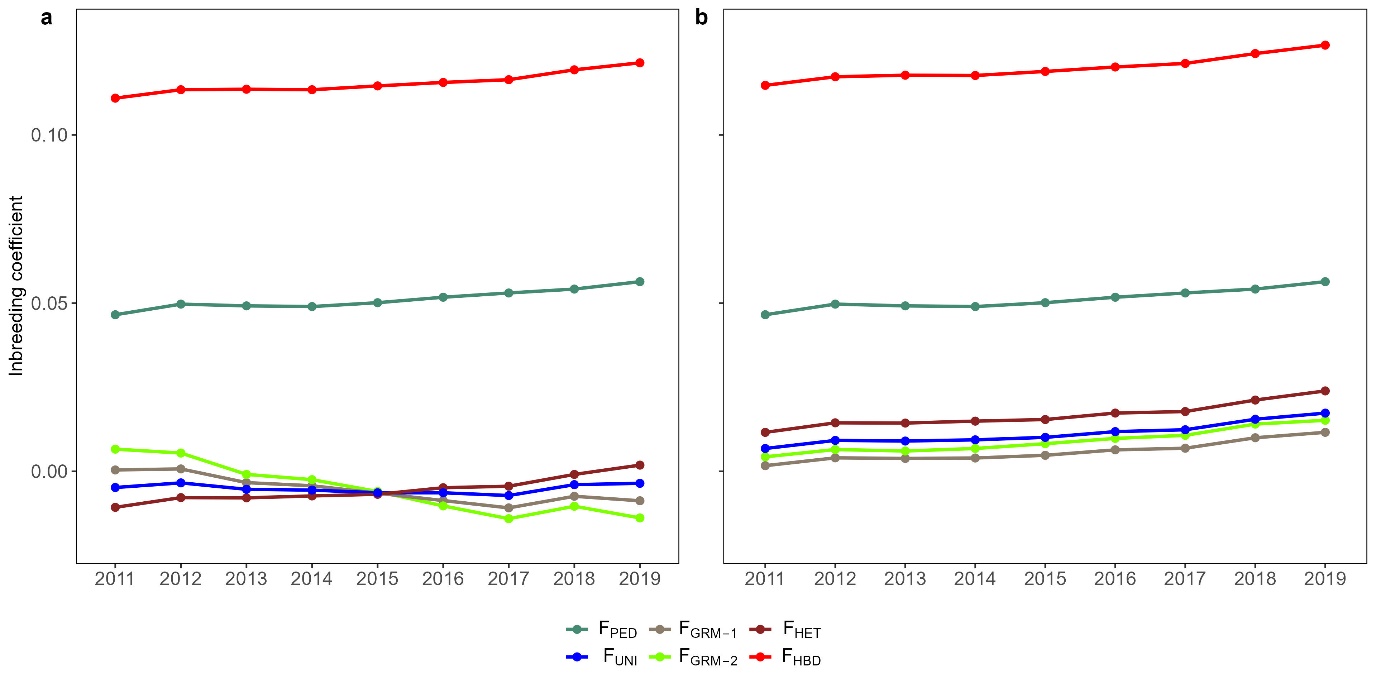


**Figure S1.** Annual trend for the average inbreeding levels by birth years (a) estimated with sample AF; (b) estimated with base population AF.


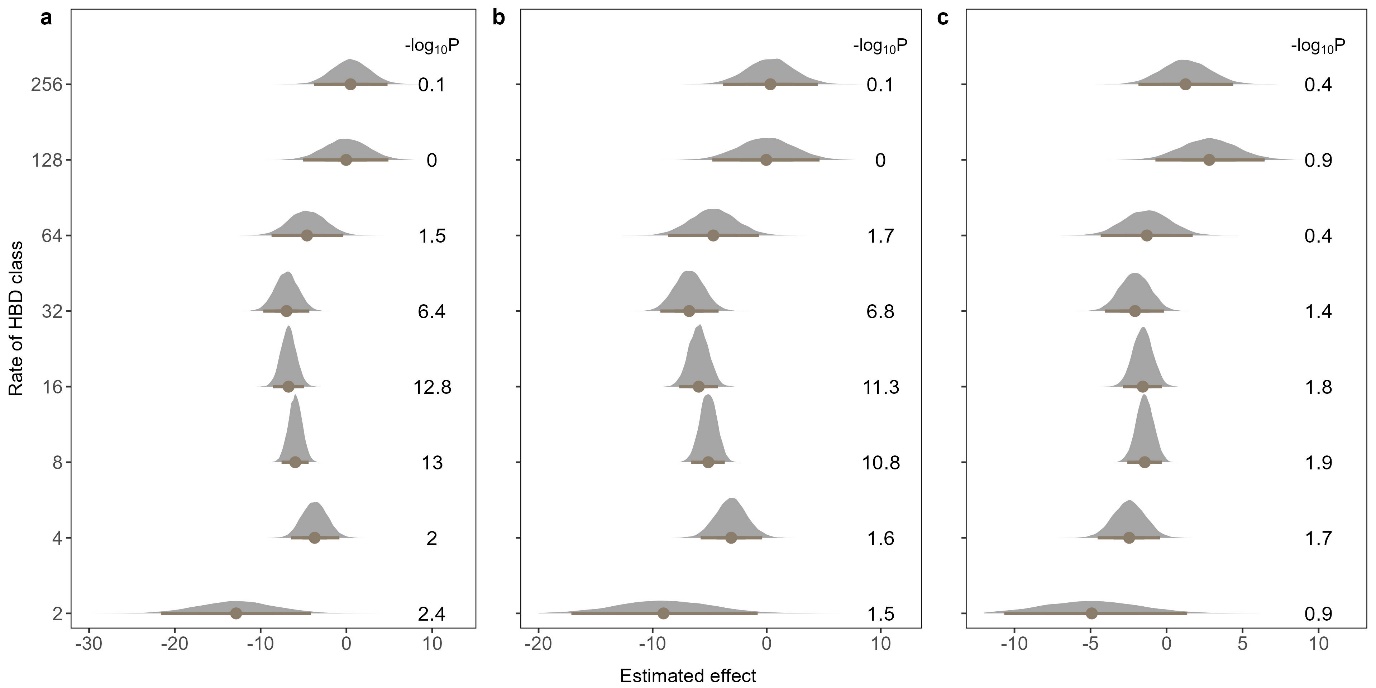


**Figure S2.** Inbreeding depression associated with different HBD classes estimated for (a) length; (b) pelvis length; (c) pelvis width. Partitioning of autozygosity in different HBD classes was performed with medium-density genotyping data (~30K SNPs). The values on the right of each panel represent the strength of association (p-value on a –log10 scale).


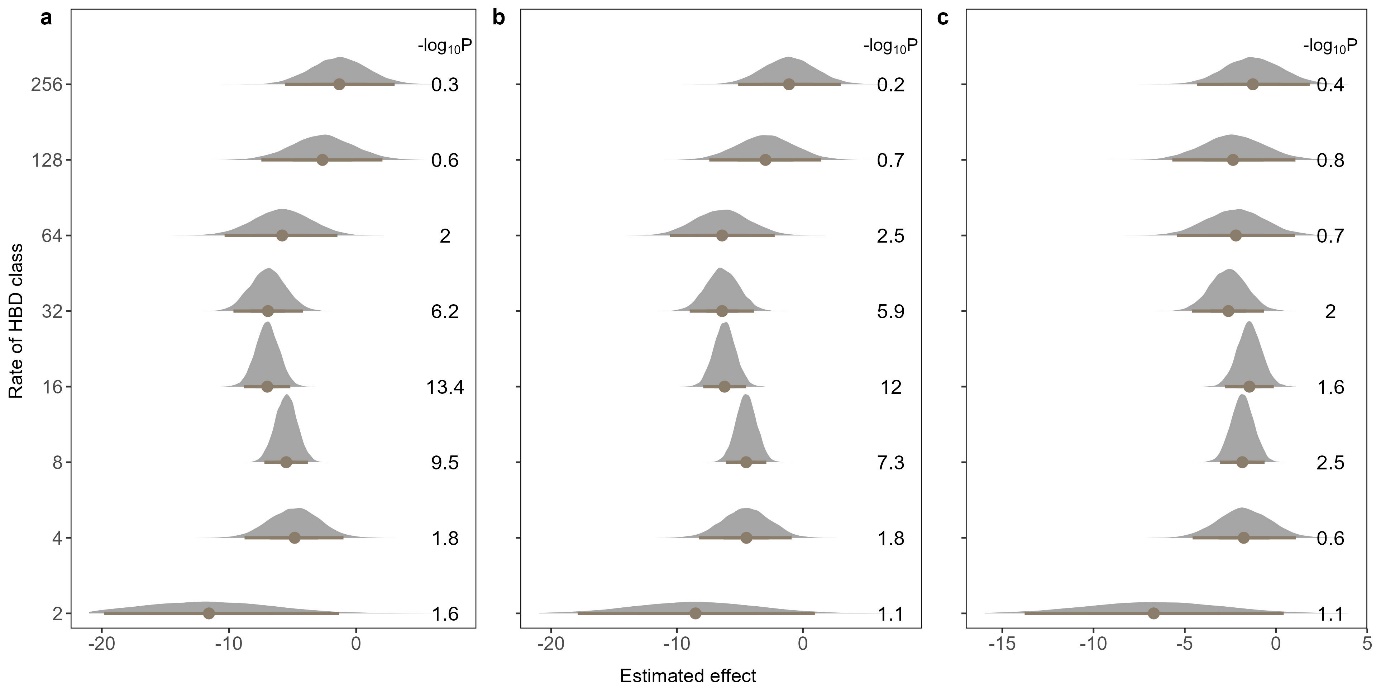


**Figure S3.** Inbreeding depression associated with different HBD classes estimated for (a) length; (b) pelvis length; (c) pelvis width. Partitioning of autozygosity in different HBD classes was performed with high-density genotyping data (~572K SNPs). The values on the right of each panel represent the strength of association (p-value on a –log10 scale).


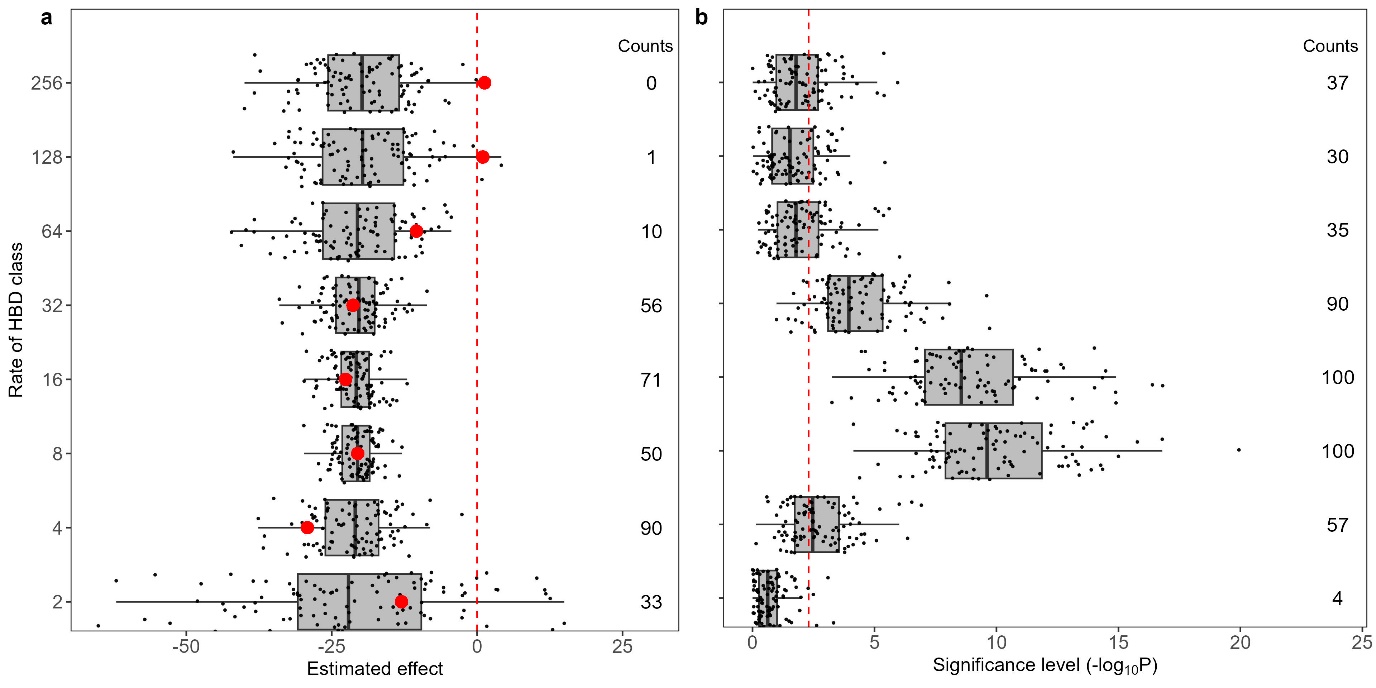


**Figure S4.** Estimated inbreeding effects (a) and associated significance levels (b) for 100 simulations. Each replicate was simulated under the assumption that all HBD classes have the same effect (-21). Partitioning of autozygosity in different HBD classes was performed with high-density genotyping data (~572K SNPs). The counts on the right of each panel represent the number of simulations with higher effects than the value estimated in the real data (panel a) or the number of simulations with significant association (panel b).


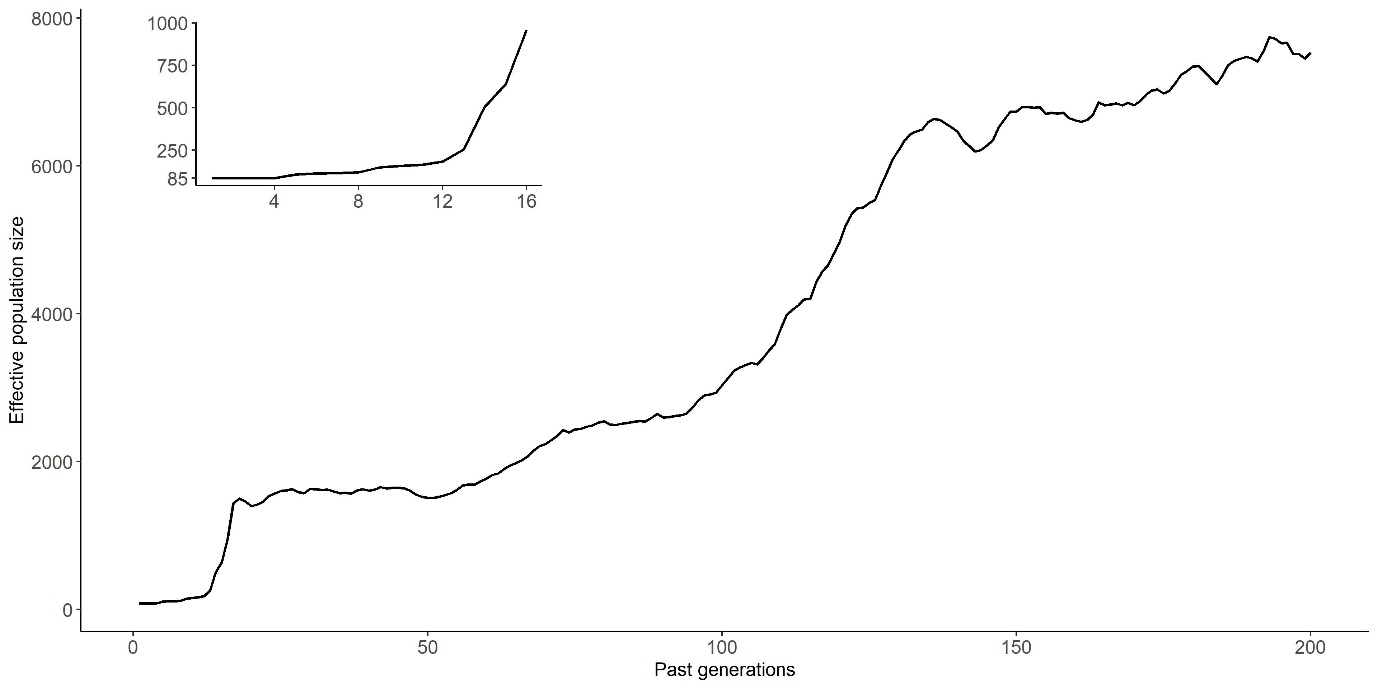


**Figure S5.** Recent evolution of past effective population size (N_e_). Effective population size was estimated using GONE [38] and with a set of 634 Belgian Blue bulls genotyped for 613,005 markers from the BovineHD genotyping array [33].
